# Bioactive 7-Oxabicyclic[6.3.0]lactam and 12-Membered Macrolides from a Gorgonian-Derived *Cladosporium* sp. Fungus

**DOI:** 10.3390/md13074171

**Published:** 2015-07-07

**Authors:** Fei Cao, Qin Yang, Chang-Lun Shao, Chui-Jian Kong, Juan-Juan Zheng, Yun-Feng Liu, Chang-Yun Wang

**Affiliations:** 1 Key Laboratory of Marine Drugs, The Ministry of Education of China, School of Medicine and Pharmacy, Ocean University of China, Qingdao 266003, China; E-Mails: caofei2016@gmail.com (F.C.); shaochanglun@ouc.edu.cn (C.-L.S.); haiyangbencao@ouc.edu.cn (C.-J.K.); zhengjuanjuan90@gmail.com (J.-J.Z.); liuyunfeng1990@gmail.com (Y.-F.L.); 2 Chinese Center for Chirality, Key Laboratory of Medicinal Chemistry and Molecular Diagnosis of Ministry of Education, and College of Pharmacy Sciences, Hebei University, Baoding 071002, China; E-Mail: transtaralice@hotmail.com

**Keywords:** gorgonian-derived fungus, *Cladosporium* sp., bicyclic lactam, 12-membered macrolides, cytotoxic activity

## Abstract

One new bicyclic lactam, cladosporilactam A (**1**), and six known 12-membered macrolides (**2**–**7**) were isolated from a gorgonian-derived *Cladosporium* sp. fungus collected from the South China Sea. Their complete structural assignments were elucidated by comprehensive spectroscopic investigation. Quantum chemistry calculations were used in support of the structural determination of **1**. The absolute configuration of **1** was determined by calculation of its optical rotation. Cladosporilactam A (**1**) was the first example of 7-oxabicyclic[6.3.0]lactam obtained from a natural source. Compound **1** exhibited promising cytotoxic activity against cervical cancer HeLa cell line with an IC_50_ value of 0.76 μM.

## 1. Introduction

**Figure 1:**
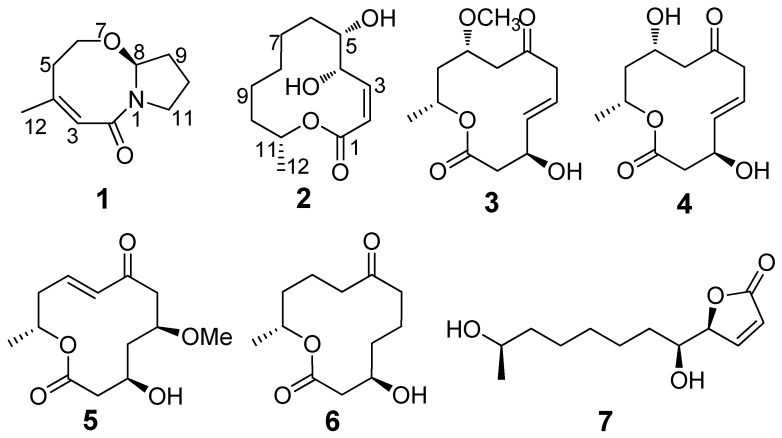
Structures of compounds **1**–**7**.

Marine fungi are known for their ability to sequester novel natural products with unique chemical scaffolds possessing a variety of potent biological activities [1]. The marine-derived *Cladosporium* fungi have been known to produce various secondary metabolites with novel structures and interesting biological activities such as cytotoxic sporiolides A and B [2], antibacterial Sumiki’s acid (5-hydroxymethylfuran-2-carboxylic acid) [3], and antifouling 3-phenyl-2-propenoic acid [4]. During our ongoing search for structurally novel and bioactive natural products from gorgonians and their symbiotic microorganisms in the South China Sea [5,6,7,8,9,10], we investigated the metabolites of 14 fungal strains cultured from the gorgonian *Anthogorgia ochracea* by integrated chemical and bioassay screening. Among them, the strain RA07-1 identified as *Cladosporium* sp. exhibited cytotoxicity and was selected for further chemical investigation. Bioassay-guided separation resulted in the isolation of a novel cytotoxic bicyclic lactam, cladosporilactam A (**1**), and six known 12-membered macrolides cladospolide B (**2**) [11], dendrodolides A, C, M, L (**3**, **4**, **5**, **6**) [12], and *iso*-cladospolide B (**7**) [13] (Figure 1). Herein we report the isolation, structure elucidation, and bioactivities of these compounds.

## 2. Results and Discussion

Cladosporilactam A (**1**) was obtained as a colorless oil. The molecular formula was determined as C_10_H_15_O_2_N on the base of positive HRESIMS, indicating four degrees of unsaturation. The ^1^H and ^13^C NMR spectra (Figures S1 and S2) (Table 1) displayed signals for one methyl group, five methylenes including an oxygen-bearing methylene and a nitrogen-bearing methylene (δ_H_ 3.54; δ_C_ 44.9), two methines including an olefinic carbon (δ_H_ 5.84; δ_C_ 121.8) and an unusual oxygen-bearing carbon (δ_H_ 5.19; δ_C_ 88.4), and two quaternary carbons including a carbonyl carbon (δ_C_ 167.5) and an olefinic carbon (δ_C_ 145.4). Analysis of the ^1^H and ^13^C NMR spectra, with the aid of HMQC spectra (Figure S3), revealed the presence of a trisubstituted double bond. These functional groups accounted for two out of the four degrees of unsaturation, and the remaining two thus required a bicyclic nucleus of **1**.

**Table 1:** NMR spectroscopic data (500/125 MHz, CDCl_3_) for compound **1**.

Position	δ_C_ Type	δ_C-pred_ *^a^* Type	δ_H_ Mult. (*J* in Hz)
2	167.5, C	165.0, C	
3	121.8, CH	126.0, CH	5.84, brs
4	145.4, C	146.9, C	
5	36.3, CH_2_	36.8, CH_2_	2.56, dd (15.0, 10.0)
			2.17, dd (15.0, 7.5)
6	66.3, CH_2_	61.2, CH_2_	4.00, dd (12.0, 7.5)
			3.43, dd (12.0, 10.0)
8	88.4, CH	87.4, CH	5.19, d (4.5)
9	34.2, CH_2_	33.7, CH_2_	1.93, m
10	21.6, CH_2_	21.9, CH_2_	2.15, m
			1.91, m
11	44.9, CH_2_	42.9, CH_2_	3.54, m
12	25.6, CH_3_	30.1, CH_3_	1.87, s

*^a^* Predicted δ_C_ for 8*R*-**1**.

**Figure 2:**
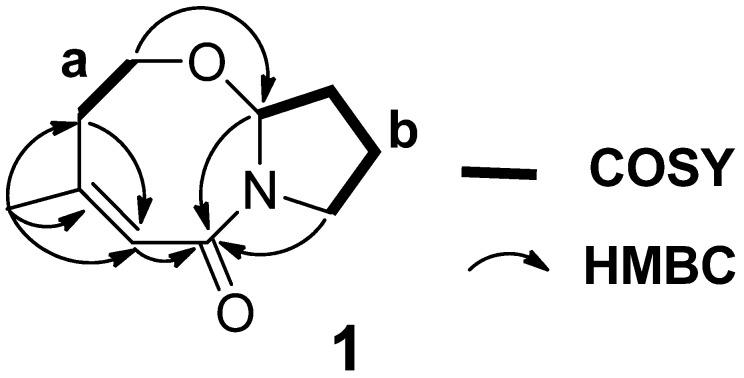
COSY and key HMBC correlations of **1**.

Extensive analysis of HMQC and COSY (Figure S4) gave two fragments (Figure 2, **a** and **b**). In order to determine the connectivity between the two partial structures, HMBC experiments (Figure S5) were carried out. The HMBC correlations (Figure 2) from H_3_-12 to C-3, C-4 and C-5, and from H_2_-5 to C-3 established the connections of C-4 with C-3, C-5 and C-12. And the HMBC correlation from H-6 to C-8 indicated the connection of C-6–O–C-8. While, both of H-8 and H_2_-11 showed HMBC cross-peaks with C-2 that suggested the amide carbonyl carbon C-2 was connected to C-8, the carbon bearing both oxygen and nitrogen, and C-11, the nitrogen-bearing carbon, via a nitrogen bridge. Thus, the planar structure of **1** was established.

NMR chemical shift calculation by quantum chemistry methods represents a powerful strategy for chemical structure interpretation [14]. To confirm the framework of **1**, the *gauge*-independent atomic orbital (GIAO) based ^13^C NMR chemical shifts calculation [15] were carried out. Conformational searches were performed using MMFF94S force field for 8*R*-**1**. All geometries with relative energy from 0 to 5.0 kcal/mol were used in optimizations at the B3LYP/6-311+G(d) level first. The minimum geometries, 8*R*-**1a** (48.6%) and 8*R*-**1b** (51.4%) (Figure S6), with relative energy from 0 to 3.0 kcal/mol were further optimized by TDDFT at the B3LYP/6-311+G(d) level. Based on the optimized geometries, the NMR calculations were performed at the B3LYP/6-311G+(2d,p) level of theory. The relative chemical shifts of 8*R*-**1a** and 8*R*-**1b** were calculated based on the chemical shifts of TMS calculated by the same procedure. All the calculations were performed using the Gaussian 09 program [16]. The individual deviations, |Δδ|, between the predicted (δ_C-pred_ of 8*R*-**1**) and experimental ^13^C chemical shifts for **1** were less than 5.1 ppm (Table 1), suggesting the δ_C_ of **1** matched the calculated δ_C_ very well. These results demonstrate that the calculations may be sufficient to confirm the framework for **1**. 

The absolute configuration at C-8 of **1** was assigned by comparing the computed optical rotation (OR) with the experimental OR. The computed OR in the gas phase for 8*R*-**1** was +70.4 at the B3LYP/6-311+G (2d,p) level. The recorded OR for **1** was −50.1 (*c* 0.80, MeOH). The absolute value of the recorded OR for **1** correspond to the computed value; however, the OR signs (negative *vs.* positive) contradict each other. These results of qualitative analysis allowed the assignment of the absolute configuration of **1** as 8*S*. 

Although bicyclic lactams have been reported from marine-derived fungi, they were mainly obtained as 1,4-diazepine [17]. In present study, the isolated compound **1** is a 7-oxabicyclic[6.3.0]lactam. Compound **1** represents the first example of bicyclic lactam characterized with the bicyclic[6.3.0]lactam nucleus structure from a natural source. The carbon framework of **1** was similar to the linear chain amide product, fuscoatramide, isolated from the fungus *Humicola fuscoatra* [18]. This raises the intriguing possibility for that **1** might derive from a linear chain amide precursor by two biological acetal reaction steps.

The cytotoxic activities of **1**–**7** were evaluated against a panel of human tumor cell lines. Compound **1** exhibited significant cytotoxicity towards cervical cancer HeLa, mouse lymphocytic leukemia P388, human colon adenocarcinoma HT-29, and human lung carcinoma A549 cell lines with the IC_50_ values of 0.76, 1.35, 2.48, and 3.11 μM, respectively.

All the isolated compounds **1**–**7** were also evaluated for antibacterial activity against a panel of pathogenic bacteria, including *Bacillus cereus*, *Tetragenococcus halophilus*, *Staphylococcus epidermidis*, *Staphylococcus aureus*, *Escherichia coli*, *Pseudomonas putida*, *Nocardia brasiliensis*, and *Vibrio parahaemolyticus* (Table 2). Compounds **3**–**5** and **7** showed antibacterial activity againstall of the pathogenic bacteria, with MIC values ranging from 3.13 to 25.0 μM. Among the active compounds, **3** and **4** exhibited the same highest antibacterial activity against *T. halophilus* (MIC = 3.13 μM).

**Table 2:** Tests of MIC (μM) for **1**–**7** against eight bacterial strains.

Strains	Compounds					
	1, 2, 6	3	4	5	7	Ciprofloxacin
*B. cereus*	>25.0	12.5	25.0	6.25	6.25	1.56
*T. halophilus*	>25.0	3.13	3.13	25.0	6.25	1.56
*S. epidermidis*	>25.0	6.25	25.0	25.0	25.0	0.78
*S. aureus*	>25.0	6.25	25.0	12.5	25.0	0.39
*E. coli*	>25.0	12.5	12.5	25.0	25.0	1.56
*P. putida*	>25.0	12.5	25.0	6.25	6.25	0.39
*N. brasiliensis*	>25.0	6.25	12.5	25.0	12.5	0.78
*V. parahaemolyticus*	>25.0	12.5	25.0	25.0	25.0	1.56

## 3. Experimental Section

### 3.1. General Experimental Procedures

The optical rotations were measured on a JASCO P-1020 digital polarimeter (JASCO Corporation, Tokyo, Japan). UV spectra were recorded using a Milton-Roy spectrophotometer (Milton Roy, New York, NY, USA). IR spectra were recorded on a Nicolet-Nexus-470 spectrophotometer using KBr pellets (Thermo Electron, Waltham, MA, USA). NMR spectra were recorded on an Agilent DD2 500 MHz NMR spectrometer (500 MHz for ^1^H and 125 MHz for ^13^C) (JEOL, Tokyo, Japan), using TMS as internal standard. ESIMS and HRESIMS spectra were measured on a Micromass Q-TOF spectrophotometer (Waters Corp., Manchester, UK) and Thermo Scientific LTQ Orbitrap XL spectrometer (Thermo Fisher Scientific, Bremen, Germany). HPLC separation was performed using a Hitachi LA-2000 prep-HPLC system (Hitachi High Technologies, Tokyo, Japan) coupled with a Hitachi L-2455 photodiodearray detector (Hitachi High Technologies, Tokyo, Japan). A Kromasil C_18_ semi-preparative HPLC column (250 mm × 10 mm, 5 μm) (Eka Nobel, Bohus, Sweden) was used. Silica gel (200–300 mesh; Qingdao Marine Chemical Group Co., Qingdao, China) and Sephadex LH-20 (Amersham Biosciences Inc., Piscataway, NJ, USA) were used for column chromatography. Precoated silica gel GF254 plates (Yantai Zifu Chemical Group Co., Yantai, China) were used for analytical TLC.

### 3.2. Fungal Materials

The fungal strain *Cladosporium* sp. (RA07-1) was isolated from a piece of fresh tissue from the inner part of the gorgonian *Anthogorgia ochracea* (GXWZ-07), collected from Weizhou coral reef in the South China Sea in April 2011. The strain was deposited in the Key Laboratory of Marine Drugs, the Ministry of Education of China, School of Medicine and Pharmacy, Ocean University of China, Qingdao, China, with the GenBank (NCBI) accession number KP720581.

### 3.3. Extraction and Isolation

The fungus *Cladosporium* sp. RA07-1 was cultivated in a rice medium in 500 mL Erlenmeyer flasks (each containing rice 80 g, water 120 mL, sea salt 2.0 g) at 27 °C for four weeks. The fermented rice substrate (40 flasks) was extracted repeatedly with EtOAc (3 × 300 mL for each flask). The combined EtOAc layer was evaporated to dryness under reduced pressure to give an EtOAc extract. The EtOAc extract (10.0 g) was subjected to column chromatography (CC) on silica gel using a step gradient elution with EtOAc–petroleum ether (PE) (0%–100%) and then with MeOH–EtOAc (0%–100%) to afford six fractions (Fr.1–Fr.6). Fr.4 was subjected to Sephadex LH-20 CC eluting with mixtures of CH_2_Cl_2_/MeOH (1:1). Then purification by semi-preparative HPLC using a C_18_ column at a flow rate of 2.0 mL/min (MeOH/H_2_O, 35:65) yielded **1** (4.3 mg). Fr.5 was separated by silica gel CC (PE/EtOAC, 1:2) and semi-preparative HPLC (MeOH/H_2_O, 40:60) to offer **2** (3.5 mg), **3** (4.0 mg), **4** (2.0 mg), **5** (3.0 mg), **6** (4.0 mg), and **7** (4.0 mg).

Cladosporilactam A (**1**): Colorless oil; [*α*]^20^_D_ −50.1° (*c* 0.80, MeOH); UV (MeOH) λ_max_ (log ε): 213 (2.89) nm; IR (KBr) *ν_max_* 2931, 2364, 1658, 1611, 1428, 1114 cm^–1^; ^1^H and ^13^C NMR data, see Table 1; ESIMS *m/z* 385.4 [2M + Na]^+^, 220.2 [M + K]^+^, 204.2 [M + Na]^+^, 182.2 [M + H]^+^ (Figure S7); HRESIMS *m/z* 182.1174 [M + H]^+^ (calcd for C_10_H_16_O_2_N, 182.1176) (Figure S8). 

Cladospolide B (**2**). White, amorphous solid; [*α*]^20^_D_ +18.7° (*c* 0.10, MeOH) [lit. 11: +26.9° (*c* 0.40, MeOH)].

Dendrodolide A (**3**). White, amorphous solid; [*α*]^20^_D_ +32.0° (*c* 0.20, MeOH) [lit. 12: +41.9° (*c* 0.37, CHCl_3_)].

Dendrodolide C (**4**). Colorless oil; [*α*]^20^_D_ +98.4° (*c* 0.20, MeOH) [lit. 12: +122° (*c* 0.09, CHCl_3_)].

Dendrodolide M (**5**). Colorless oil; [*α*]^20^_D_ +85.8° (*c* 0.20, MeOH) [lit. 12: +92.6° (*c* 0.105, CHCl_3_)].

Dendrodolide L (**6**). White, amorphous solid; [*α*]^20^_D_ +23.6° (*c* 0.20, MeOH) [lit. 12: +10.7° (*c* 0.065, CHCl_3_)].

*iso*-Cladospolide B (**7**). White, amorphous solid; [*α*]^20^_D_ −106.2° (*c* 0.20, MeOH) [lit. 13: −90° (*c* 0.23, MeOH)].

### 3.4. Biological Assays

The cytotoxic activities of **1**–**7** against mouse lymphocytic leukemia P388 and human lung carcinoma A549 cell lines were evaluated using the SRB method [19]. The cytotoxic activity against cervical cancer HeLa and human colon adenocarcinoma HT-29 cell lines were evaluated using the MTT method [20]. Adriamycin was used as a positive control.

Antibacterial activities were evaluated by the conventional broth dilution assay [21]. Eight pathogenic bacterial strains, *Bacillus cereus*, *Tetragenococcus halophilus*, *Staphylococcus epidermidis*, *Staphylococcus aureus*, *Escherichia coli*, *Pseudomonas putida*, *Nocardia brasiliensis*, and *Vibrio parahaemolyticus* were used, and ciprofloxacin was used as a positive control.

## 4. Conclusions

In summary, a bicyclic lactam, cytotoxic 7-oxabicyclic[6.3.0]lactam (**1**) has been isolated from a gorgonian-derived *Cladosporium* sp. fungus collected from the South China Sea. The structure of **1** was confirmed by quantum chemistry calculation methods. Compound **1** showed cytotoxic activity against HeLa, P388, HT-29 and A549 cell lines, suggesting that it might have potential to be developed as an antitumor agent.

## Supplementary Files

Supplementary File 1
