# Supplementary material for: Bioactive 7-Oxabicyclic[6.3.0]lactam and 12-Membered Macrolides from a Gorgonian-Derived Cladosporium sp. Fungus
Source: Mar Drugs. 2015 Jul 7;13(7):4171–8. doi: 10.3390/md13074171 (PMC4515610; doi:10.3390/md13074171)
Supplement: Supplementary File 1 [file marinedrugs-13-04171-s001.doc]

Supplementary Information

**Figure S1.** ^1^H NMR (500 MHz, CDCl_3_) spectrum of compound **1**.

**Figure S2.** ^13^C NMR (125 MHz, CDCl_3_) spectrum of compound **1**.

**Figure S3.** HMQC (CDCl_3_) spectrum of compound **1**.

**Figure S4.** COSY (CDCl_3_) spectrum of compound **1**.

**Figure S5.** HMBC (CDCl_3_) spectrum of compound **1**.

**Figure S6.** ESIMS spectrum of compound **1**.

**Figure S7.** HRESIMS spectrum of compound **1**.

**Figure S8.** Lowest energy 3D conformers of **8*R*-1a** (48.6%) and **8*R*-1b** (51.4%).


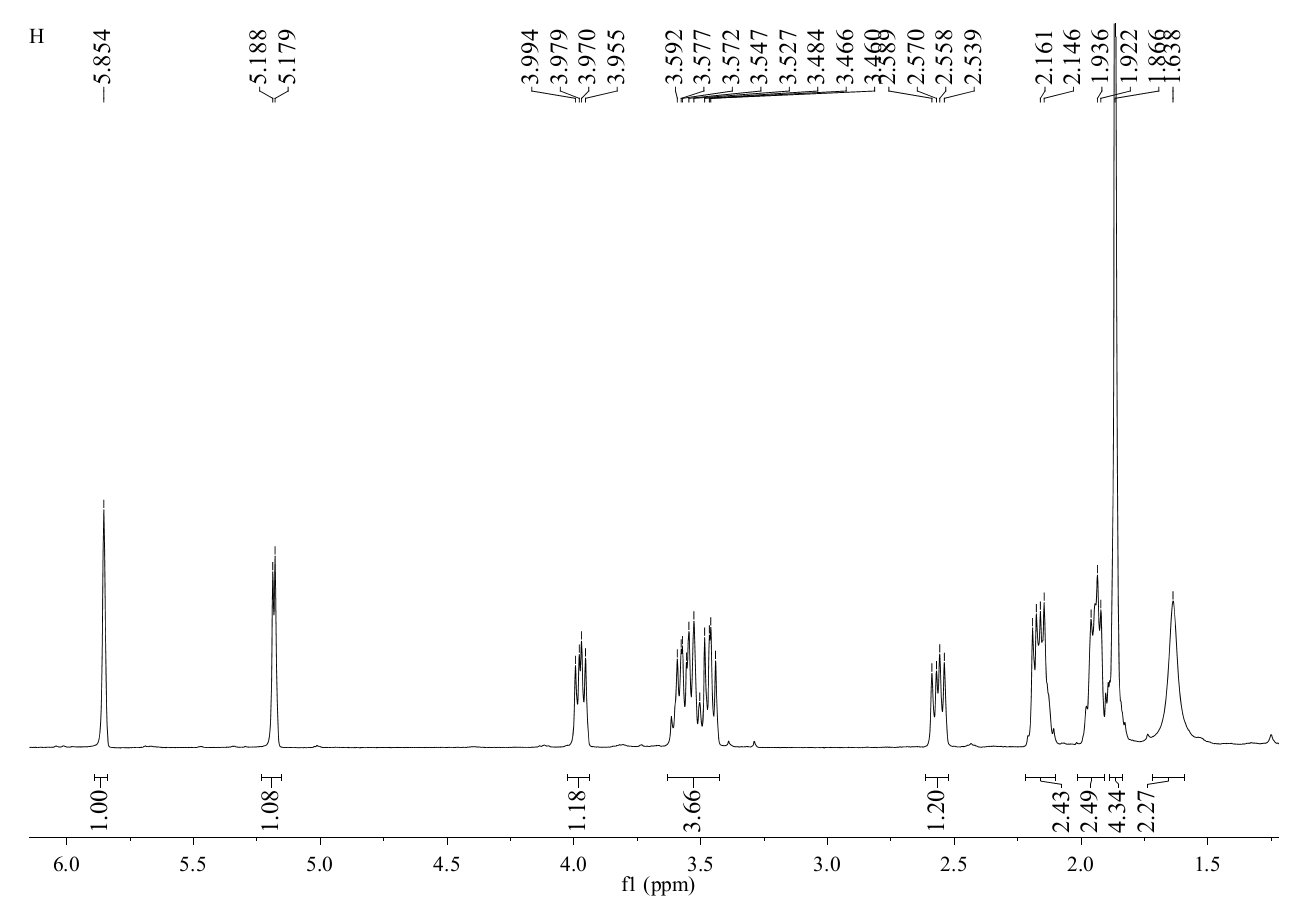


**Figure S1.** ^1^H NMR (500 MHz, CDCl_3_) spectrum of compound **1**.


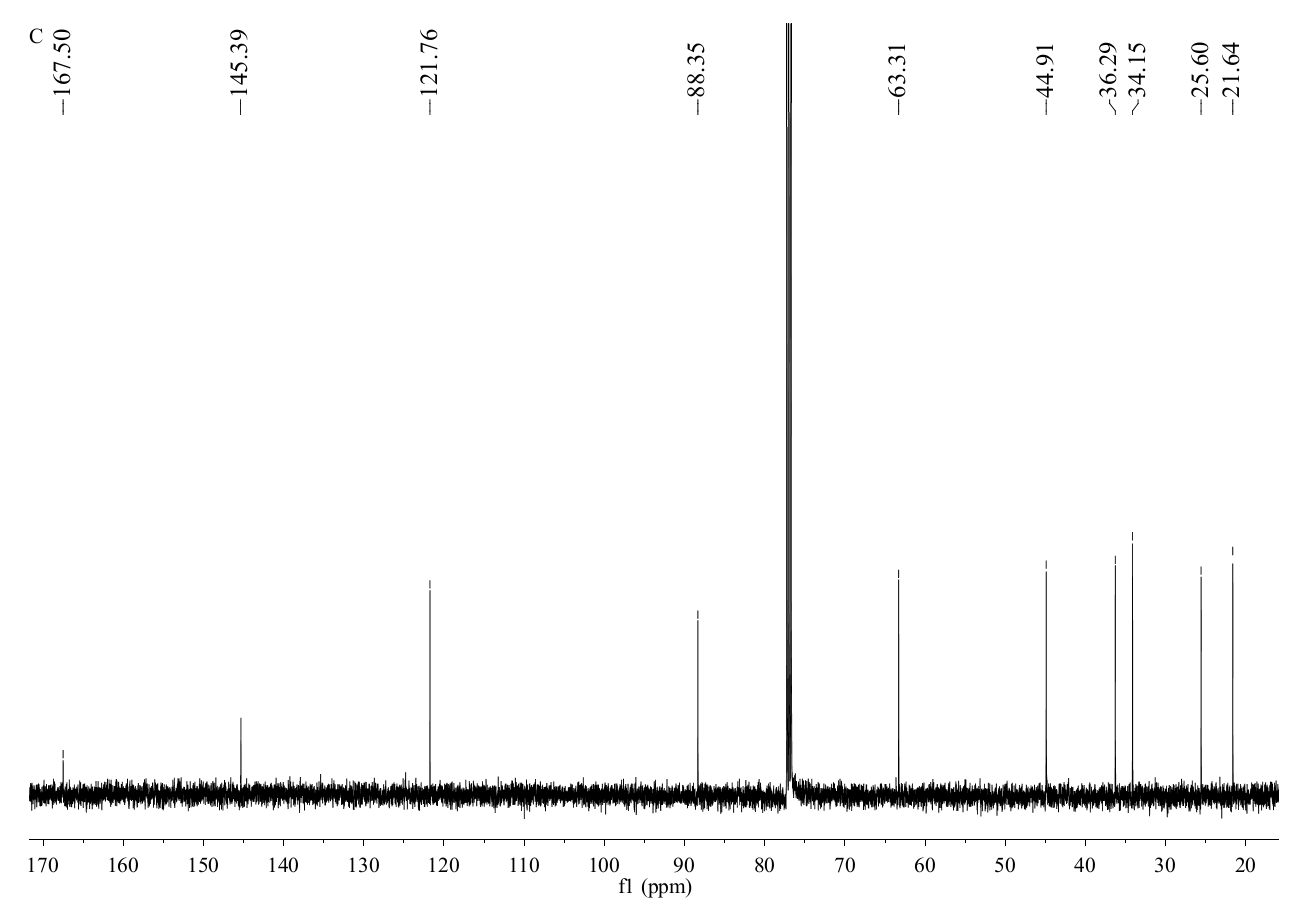


**Figure S2.** ^13^C NMR (125 MHz, CDCl_3_) spectrum of compound **1**.
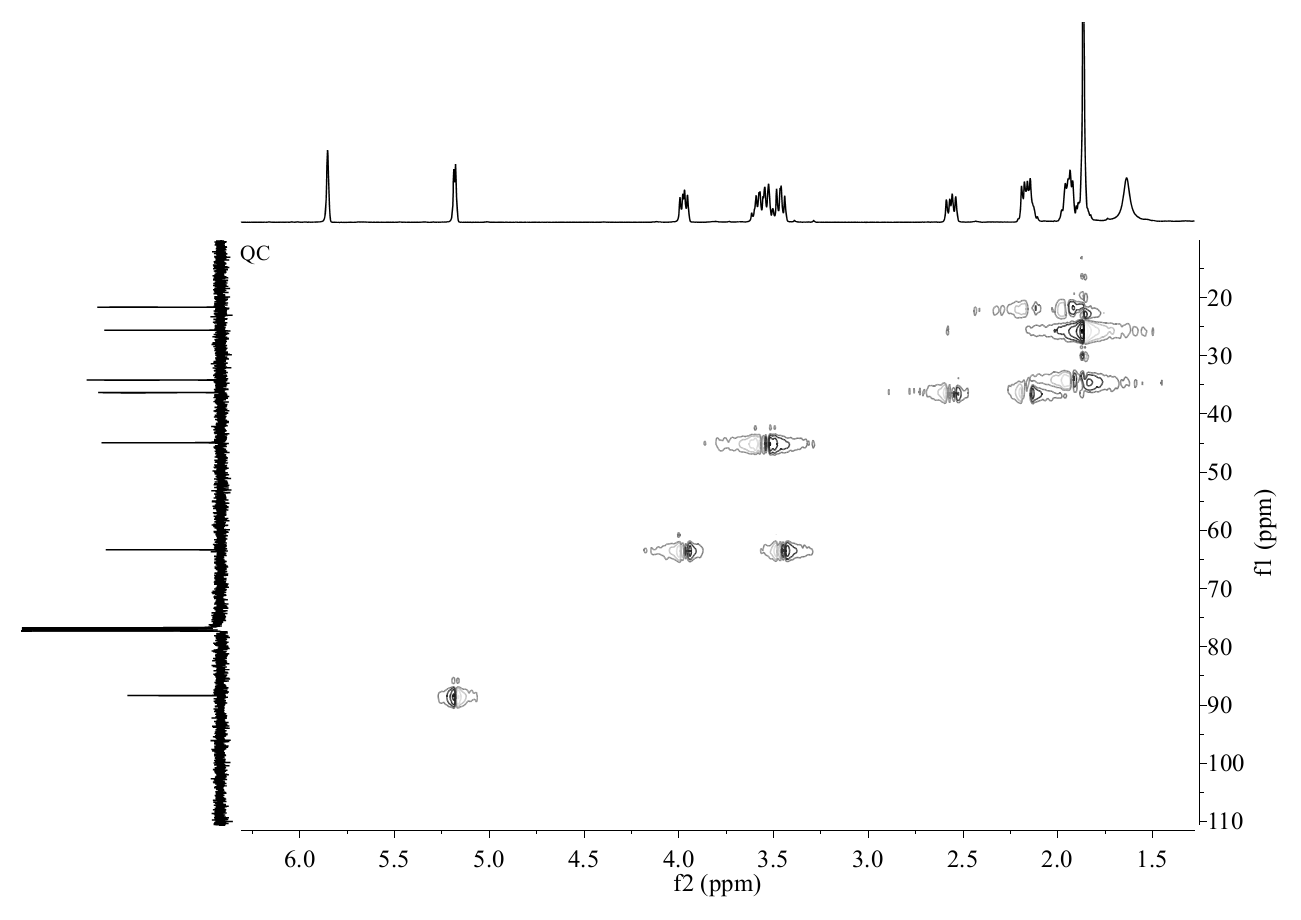


**Figure S3.** HMQC (CDCl_3_) spectrum of compound **1**.


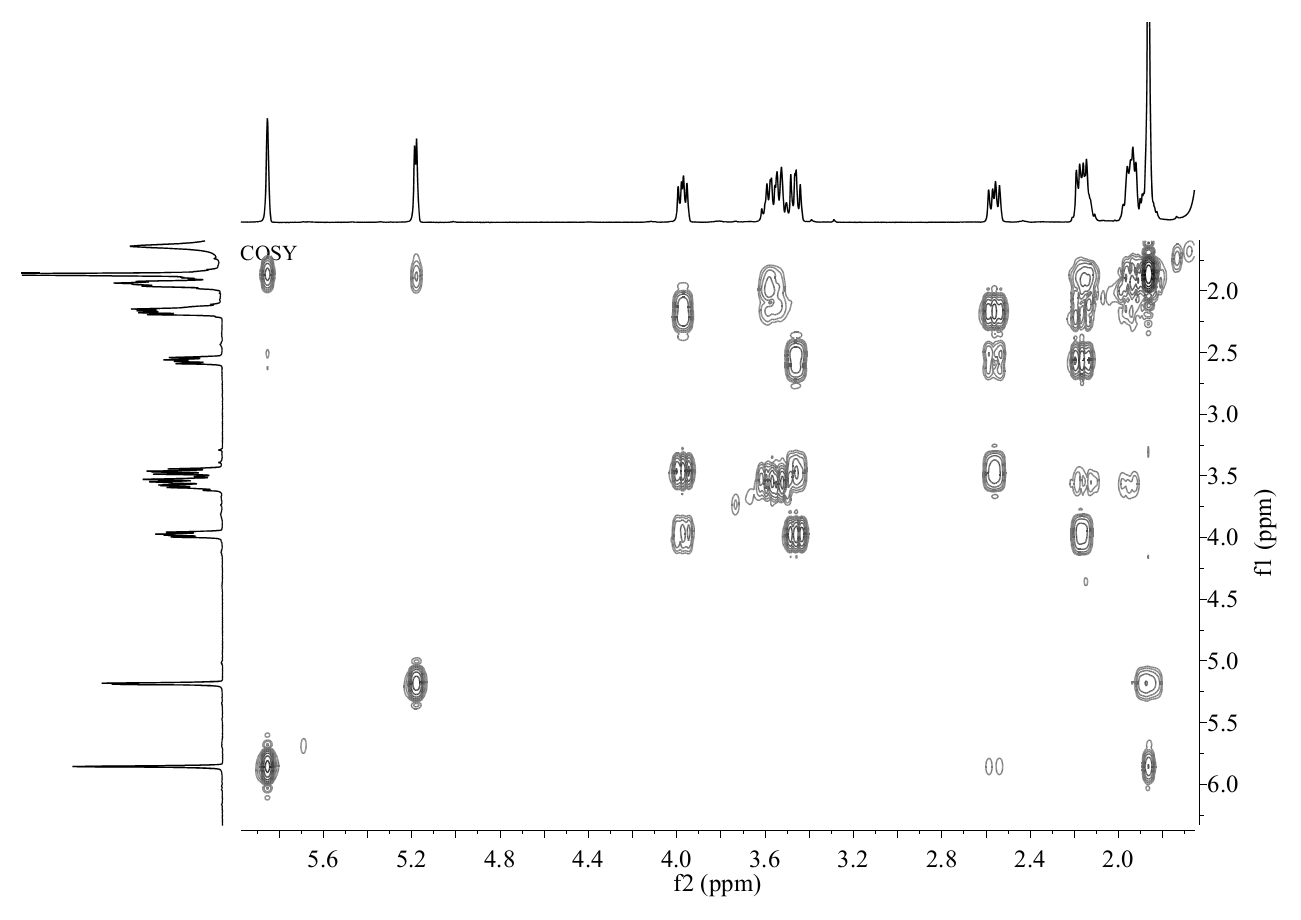


**Figure S4.** COSY (CDCl_3_) spectrum of compound **1**.


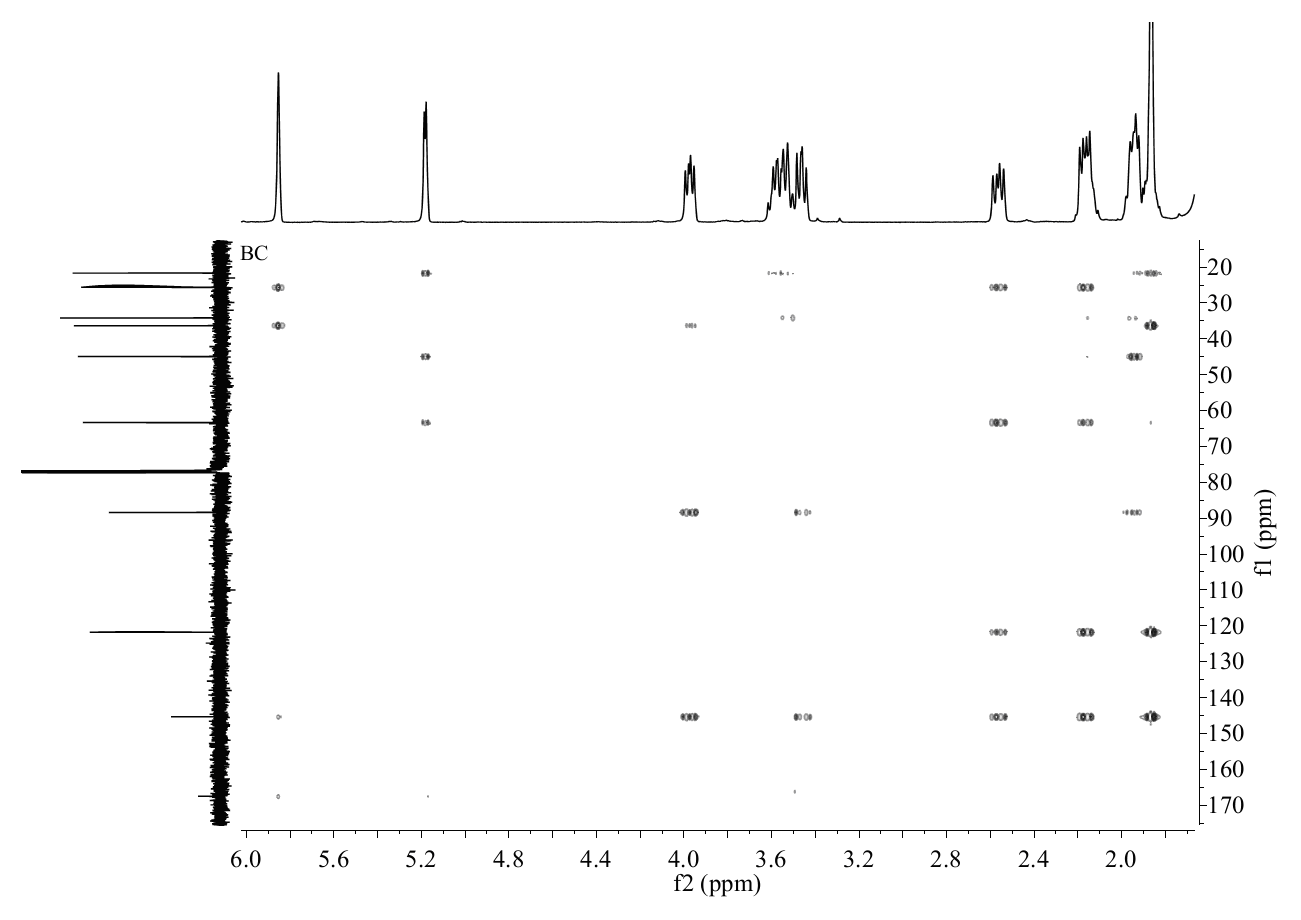


**Figure S5.** HMBC (CDCl_3_) spectrum of compound **1**.

**Figure S6.** Lowest energy 3D conformers of **8*R*-1a** (48.6%) and **8*R*-1b** (51.4%).


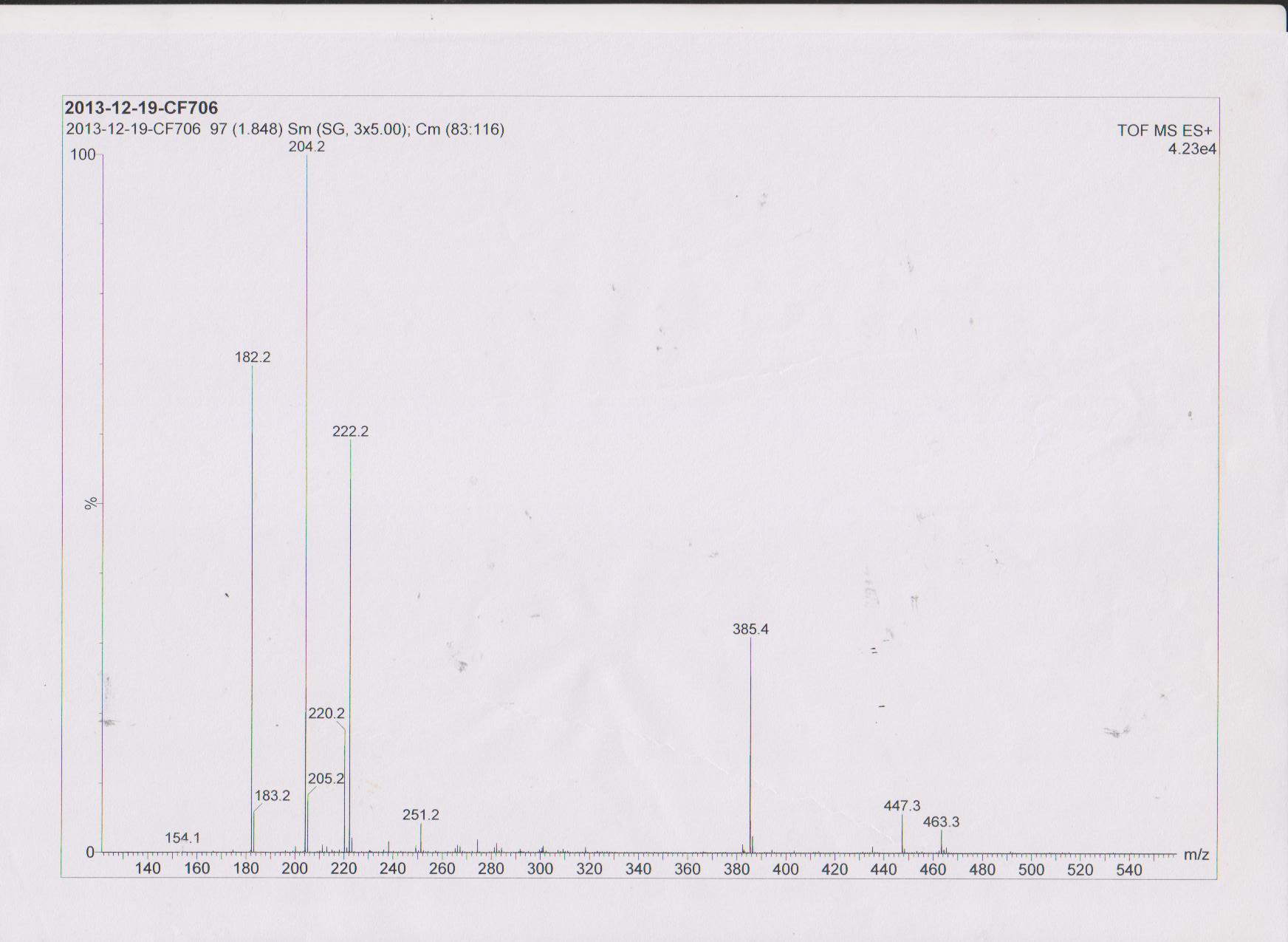


**Figure S7.** ESIMS spectrum of compound **1**.


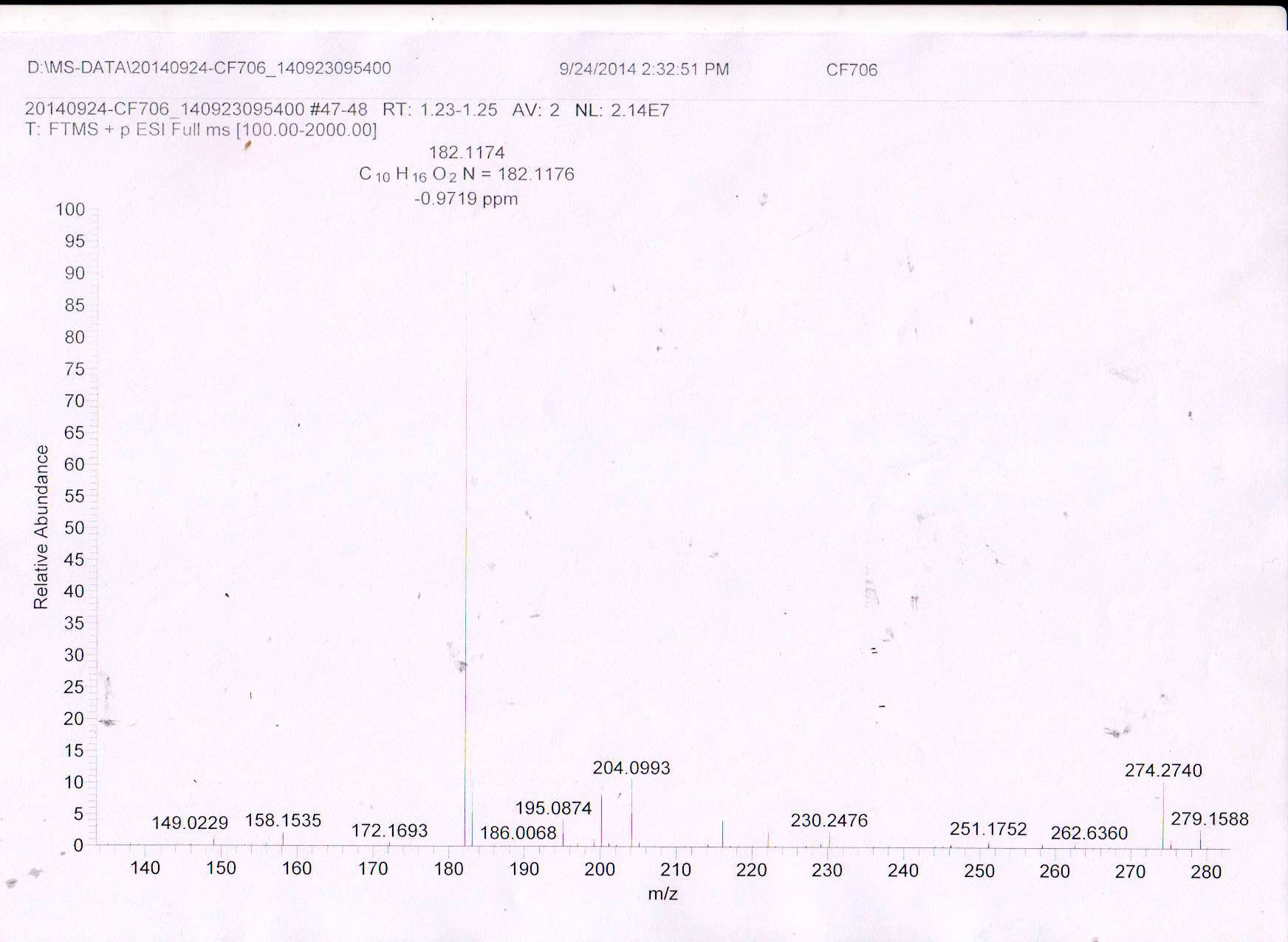


**Figure S8.** HRESIMS spectrum of compound **1**.
